# Post-encoding positive emotion impairs associative memory for English vocabulary

**DOI:** 10.1371/journal.pone.0228614

**Published:** 2020-04-06

**Authors:** Chengchen Li, Lin Fan, Bo Wang

**Affiliations:** 1 School of Foreign Languages, Huazhong University of Science and Technology, Wuhan, China; 2 National Research Center for Foreign Language Education, Beijing Foreign Studies University, Beijing, China; 3 Department of Psychology, Central University of Finance and Economics, Beijing, China; Kochi University of Technology, JAPAN

## Abstract

There is evidence that emotion induced during encoding impairs associative memory (e.g., Bisby, Horner, Bush, & Burgess, 2018), yet the effect of post-encoding emotion (particularly positive emotion) on associative memory remains largely unclear. Two experiments were conducted to examine the effect of post-encoding positive emotion on associative memory for English vocabulary. In Experiment 1, high school students memorized Chinese definitions of a list of English words, immediately recalled the Chinese definitions, watched a neutral or comic video, and took a delayed memory test 25 minutes after encoding. The result showed a significant impairing effect of post-encoding positive emotion on memory for Chinese definitions. In Experiment 2, primary school students encoded English words with their associative pictures, took an immediate test where, on each trial, they were asked to choose the correct English word that matches a picture. Following the test, they watched a neutral or comic video, and took a memory test 10 minutes after encoding. Consistent with Experiment 1, Experiment 2 showed an impairing effect of positive emotion. Taken together, these findings support the hypothesis that post-encoding positive emotion can impair associative memory, providing important implications for acquisition of vocabulary of English as a foreign language.

## Introduction

Episodic memory consists of two components: item memory and associative memory, with the former referring to the form of memory that provides the basis for remembering that a stimulus or event has been encountered, and the latter referring to the form of memory that represents relationships among items [1]. Item memory can be tested by free recall or recognition [e.g., 2,3]. Associative memory can be tested by recalling a target item in a paired associate. For instance, during encoding participants are usually presented with a series of word-pairs (e.g., bulb-pencil). For memory test they can be presented with the first word of previously learned word pairs (e.g., bulb) and asked to recall the word that completes the pair [e.g., 4]. Prior studies have shown that emotion can enhance item memory, yet the effect of emotion on associative memory remains poorly understood. Although a limited number of studies have shown that emotion induced during encoding impairs associative memory [e.g., 5], it is unclear whether post-encoding emotion (particularly positive emotion) affects associative memory, which motivates the present study.

### Effects of emotion on item memory and associative memory

There has been increasing evidence showing that emotion has differential effects on item memory and associative memory. In most studies, emotion has been found to enhance item memory [e.g., 6–11] but impair associative memory [e.g., 5, 12–16]. For instance, in a study by[16], participants learned visual objects embedded in the periphery of negative or neutral pictures. The results showed better recognition memory for negative than for neutral pictures; however, memory for the association between visual objects and pictures was worse when the pictures were negative than when they were neutral, suggesting an impairment effect of negative emotion on associative memory. According to [16], such an impairment effect may be explained by the mechanism via which emotional pictures capture attention that would otherwise be allocated to the peripheral visual objects, thus lowering the performance in a memory task that requires knowledge of the visual objects. The impairing effect was replicated in a recent study by [13], who, in three experiments, found that negative emotion induced by a negative visual stimulus or by an anticipatory threat of shock impaired associative memory. Taken together, the above studies provide converging evidence that negative emotion can impair associative memory.

### Research gaps

Despite the above findings on the effect of emotion on episodic memory, some questions remain to be answered. First, most previous empirical studies adopted a paradigm whereby learning stimuli function as the source of emotion, and emotion is thus manipulated during encoding [e.g.,^17^]. By contrast, little has been known about the effect of post-encoding emotion on episodic memory. As empirically revealed in a recent study by [18], the effect of acute stress on associative and item memory differ throughout the memory cycle (e.g., before encoding, after encoding, or before retrieval). Thus, examining the effect of post-encoding emotion contributes to a complete understanding of the effect of emotion throughout different time scales of the memory cycle and to the establishment of a refined theory for the effect of emotion on episodic memory.

Second, although post-encoding emotion has been shown to affect item memory [e.g., 2,7], its effect on associative memory has been rarely examined. The theoretical account for the results is that emotion leads to the release of a variety of substances including norepinephrine, which, along with the activation of the amygdala, modulates hippocampus-dependent memory [19]. However, support for this theoretical account has primarily come from studies using item memory tasks, and it is thus necessary to test this theoretical account using associative memory tasks. Furthermore, prior studies have suggested that post-encoding emotion may be used as a strategy of enhancing memory in educational settings [2,20]. However, if it turns out that post-encoding emotion impairs associative memory, it is necessary to be cautious when employing emotion as an intervention strategy. Therefore, it is of practical significance to understand the effect of *post-encoding* emotion on associative memory.

Third, in prior studies, negative stimuli were typically used to induce emotion [e.g., 21], leaving unclear whether positive emotion has a similar effect. According to the circumplex model proposed by [22], valence is an important dimension of emotion. In studies in which positive emotion was indeed induced after encoding, however, the dependent variable was typically item memory (i.e., recall or recognition), leaving unknown the effect of post-encoding positive emotion on associative memory. Given the evidence suggesting the role of emotional valence [e.g., 6,17], it is of critical importance to examine the effect of post-encoding positive emotion on associative memory so as to have a comprehensive understanding of the effect of emotion.

Fourth, in prior studies on the effect of post-encoding emotion, participants were undergraduates. The findings based on undergraduates, though providing important implications, may not be sufficient for theoretical building given that age is an important variable in emotional memory. Indeed, children and adolescents may be differentially subject to the impact of emotion, as evidenced by [23], who found higher memory accuracy for negative than positive words in adolescents (12-14-year-olds). However, for children (7-8-year-olds), no influence of word valence was found. Another study by [24] has also indicated that the effect of emotion can vary depending on age of participants: Although older and younger participants did not differ on recall of positive stories, older participants had lower recall of negative and neutral stories. Taken together, these studies suggest the importance of examining the effect of emotion on participants of different ages.

### Overview of the current study

Given the above research gaps, the current study was aimed at examining the effect of post-encoding positive emotion on associative memory. Although this question has not been directly addressed, studies have examined the effect of post-encoding emotion on separate elements of memory. For instance, [25] examined the effect of post-encoding emotion on separate aspects of stimulus representation. They found that negative emotion elicited after encoding enhanced memory for the gist, rather than details of stimuli. Another study by [2] showed that the effect of post-encoding emotion was dependent on the specific element of memory (at least for female participants): Negative emotion enhanced item memory (i.e., recognition memory for words) but had little effect on source memory (i.e., memory for font colors of words). Although it may be inappropriate to regard source memory and associative memory as the same, these findings suggest that post-encoding emotion may not enhance memory that involves retrieval of details. Another study by [26], in which participants experienced stress due to skydiving after encoding, showed that stress enhanced familiarity-based recognition rather than recollection for male participants. Together, results from the above studies indicate that post-encoding emotion has little effect on memory that requires retrieval of details. Associative memory, involving the retrieval of relations between items, may depend on retrieval of details of items; therefore, it is possible that post-encoding emotion would not enhance associative memory.

Particularly, there has been evidence that emotion induced during encoding can impair associative memory [13,16]. If the effect of emotion induced after encoding is similar to the effect of emotion induced during encoding, it can be hypothesized that post-encoding positive emotion would not enhance associative memory. Two experiments were conducted to test the above hypothesis. In Experiment 1, during the learning phase, high school students were presented with a number of pairs consisting of English words and their Chinese definitions. They were asked to memorize the Chinese definition of each English word. Following the initial learning, they took an immediate test, after which they watched a neutral or comic video clip, performed some filler tasks and took a delayed memory test. The task was similar to that used in a study by [27], where during the learning session participants learned the Japanese–English paired associates and during the testing session they were asked to type the English counterparts for the previously seen Japanese words. Experiment 2 was similar to Experiment 1 except that participants were primary school students, who were asked to memorize English words associated with their corresponding pictures, and took a memory test in which they were asked to choose an English word that matched a specific picture.

## Experiment 1

### Method

#### Ethics statement

Verbal informed consent was obtained from all participants and their teachers. Prior to participation, all participants were informed of the procedure, duration, and reward of the research. They were also informed that the participation was voluntary and they could withdraw at any time during the experiment. Data were collected and analyzed anonymously. The experiment was exempt from ethical review from the Institutional Review Board of School of Sociology and Psychology at Central University of Finance and Economics.

#### Participants

A sample size estimation was calculated using G*Power Version 3.1.9.2 software. Using moderate parameters (power = 0.8, effect size f = 0.25), the analysis provided an estimate of a sample size of 34. A total of 63 high school students (33 male participants and 30 female participants; mean age = 16.48 years, *SD* = .74 year) from the Affiliated School of Beijing Institute of Education took voluntary participation in the experiment. This sample size yielded a power close to 0.97. All participants were native speakers of Chinese who had about 6–7 years of English learning experience.

#### Stimuli

A total of 22 English nouns were selected as learning stimuli, two of them used in the practice phase, and the remaining twenty of them used in the formal learning phase, during which two words were placed at the beginning and the end of the learning list respectively to buffer primacy and recency effects. The remaining 16 words were presented in S1 Appendix. The selection of words was primarily based on two criteria: 1) novelty (i.e., the words are new to participants so as to allow for the examination of the effect of emotion); 2) single definition (i.e., each word has a single definition rather than multiple definitions).

Two video clips were used respectively for the control and positive groups. The neutral video for the control group was about how to repair a CD-ROM drive, and the video for the positive group was a short play by three comedians. These videos have been validated in prior studies [2,28].

#### Design and procedure

A between-subjects design was employed, with emotion group (control and positive) being the independent variable. The primary dependent variables were memory scores in the immediate and delayed tests. Participants were randomly assigned to two emotion groups. The steps and their duration were presented in Table 1.

**Table 1:** The steps and their duration in Experiment 1.

Step	Content	Duration in seconds	Duration in minutes
1	Initial knowledge test	96	1.60
2	Practice block (2 English-Chinese pairs)	32	0.53
3	Practice test	22	0.37
4	Formal learning block	320	5.33
5	Immediate test	176	2.93
6	Math task	300	5.00
7	Pre-video mood and arousal ratings	8	0.13
8	Video presentation	180	3.00
9	Post-video mood and arousal ratings	8	0.13
10	Filler tasks (math tasks and questionnaires)	1074	17.90
11	Pre-test mood and arousal ratings	8	0.13
12	Delayed test	176	2.93

The duration of the steps for tests (i.e., initial knowledge test, practice test, immediate test, and delayed test) was based on estimations because each trial did end until participants made a response. We did, however, encouraged them to type down their answers within seven seconds in the immediate and delayed tests in order to reduce temporal variations across participants.

After arriving at the laboratory, participants first took an initial knowledge test at which English words were successively presented on the screen as learning stimuli. The purpose of this test was to ascertain whether participants had already known the definitions of the English words selected as the memoranda for the experiment. For each word, participants were instructed to type its corresponding Chinese definition into a dialogue box. Then, for the practice block, two English words with their Chinese definitions were presented. Participants were informed that there would be a subsequent memory test during which they would be required to recall the Chinese definitions for their corresponding English words. On each trial, participants first saw a fixation cross at the center of a screen for one second. Then an English word accompanied by its Chinese definition in white was presented for seven seconds, against a black screen background, followed by a blank black screen lasting for one second. Afterwards, participants took the test for the English-Chinese pairs presented in the practice block.

Following the practice test, they entered the formal learning phase, during which a total of 20 English words along with their Chinese definitions were presented, with two English words at the beginning and two English words at the end of the list to buffer primacy and recency effects. The trial procedure was the same as that in the practice phase. Participants were told that their memory for definitions of the English vocabulary would be tested. Additionally, to avoid possible floor effects, participants were instructed to learn twice the list of English words. There was no learning criterion used and they were simply instructed to undergo two learning blocks (with words randomized in each block).

At the immediate test following the phase of formal learning, the 16 English words (excluding the four words used to buffer primacy and recency effects) that participants had memorized were randomly presented at the center of a screen for four seconds. After a word disappeared from the screen, participants were asked to type its corresponding Chinese definition. They were encouraged to finish the typing within seven seconds, and they were allowed to press the “Enter” key to continue, when they were unable to recall a Chinese definition. For an answer to be identified as correct, it must be exactly the same one that was studied.

After the immediate test, they performed some mathematical tasks until 8 minutes had elapsed from the end of formal learning. Then they rated their current mood and arousal on two scales ranging from 0 to 8 (with 0 representing extremely unhappy and calm, and 8 representing extremely happy and aroused). After the ratings, the control and positive groups watched a 3-minute neutral and positive video clip, respectively. At the end of video presentation, they retrospectively rated their mood and arousal during video presentation.

Participants undertook some mathematical tasks and filled in some questionnaires including arousal predisposition scale [29], emotion appraisal and emotion suppression scales [30], as well as state and trait anxiety inventory [31] until the start of the delayed memory test, which took place 25 minutes after the end of formal learning.

Before the phase of memory test, they first rated their current mood and arousal and then typed down the Chinese definitions of the 16 English words that appeared in formal learning. Each word was presented on the screen for four seconds and there was no time limit for their responses. The next word did not appear until after they pressed the “Enter” key.

#### Statistical analyses

**A**ll analyses were carried out using SPSS 13.0. The significance value was set at p < .05. Data of two participants were not successfully collected due to computer malfunction. Degrees of freedom were adjusted where equality of variances was violated. Memory performance was determined as percentage correct (i.e., correctly recalled Chinese definitions divided by the total number of encoded Chinese definitions). Outliers were excluded using the following criteria. Mild outliers are scores outside 1.5*IQR from the rest scores, with IQR representing “Interquartile range” (the middle 50% of the scores). Extreme outliers are any scores outside 3*IQR from the rest scores. The above criteria led data of five participants to be detected as outliers and excluded from analyses. The final analyses were conducted on data of 56 participants (27 and 29 participants in the control and positive groups, respectively).

For manipulation check on emotion elicitation, a 2 (time: before video presentation vs. after video presentation) × 2 (emotion group: control vs. positive) repeated-measures ANOVA was conducted on mood and arousal ratings, respectively. For evaluating the effect of emotion on memory performance, a 2 (time: immediate vs. delayed) × 2 (emotion group: control vs. positive) repeated-measures ANOVA was conducted on memory scores in the immediate and delayed tests. This method of analyses is in accord with prior studies [e.g., 32,33] and with the suggestion proposed by Dr. Lee A. Becker at University of Colorado (https://www.uccs.edu/lbecker). Using the repeated measures ANOVA has the advantage enabling the understanding of how memory changes differentially over time in the two emotion groups.

#### Results

*Participants’ characteristics*. Table 2 presents participants’ characteristics as reflected from the questionnaires. It can be seen that the two groups did not significantly differ in the five characteristics. Therefore, memory differences between the control and positive groups cannot be attributed to the differences in these characteristics.

**Table 2:** Participants' characteristics in the control and positive groups in Experiment 1.

Measure	Control (n = 27)		Positive (n = 29)		*p*
	*M*	*SD*	*M*	*SD*	
Arousal predisposition	32.41	6.65	33.10	8.11	.73
Emotion regulation	27.48	5.32	27.17	4.92	.82
Emotion suppression	15.81	4.33	15.90	4.38	.94
Trait anxiety	45.85	9.87	46.72	9.84	.74
State anxiety	44.78	7.28	47.28	7.86	.22
Age	16.41	0.75	16.48	0.69	.70
M/F	13/14		15/14		.79

M/F represents the ratio of number of male participants to the number of female participants.

**Fig 1:**
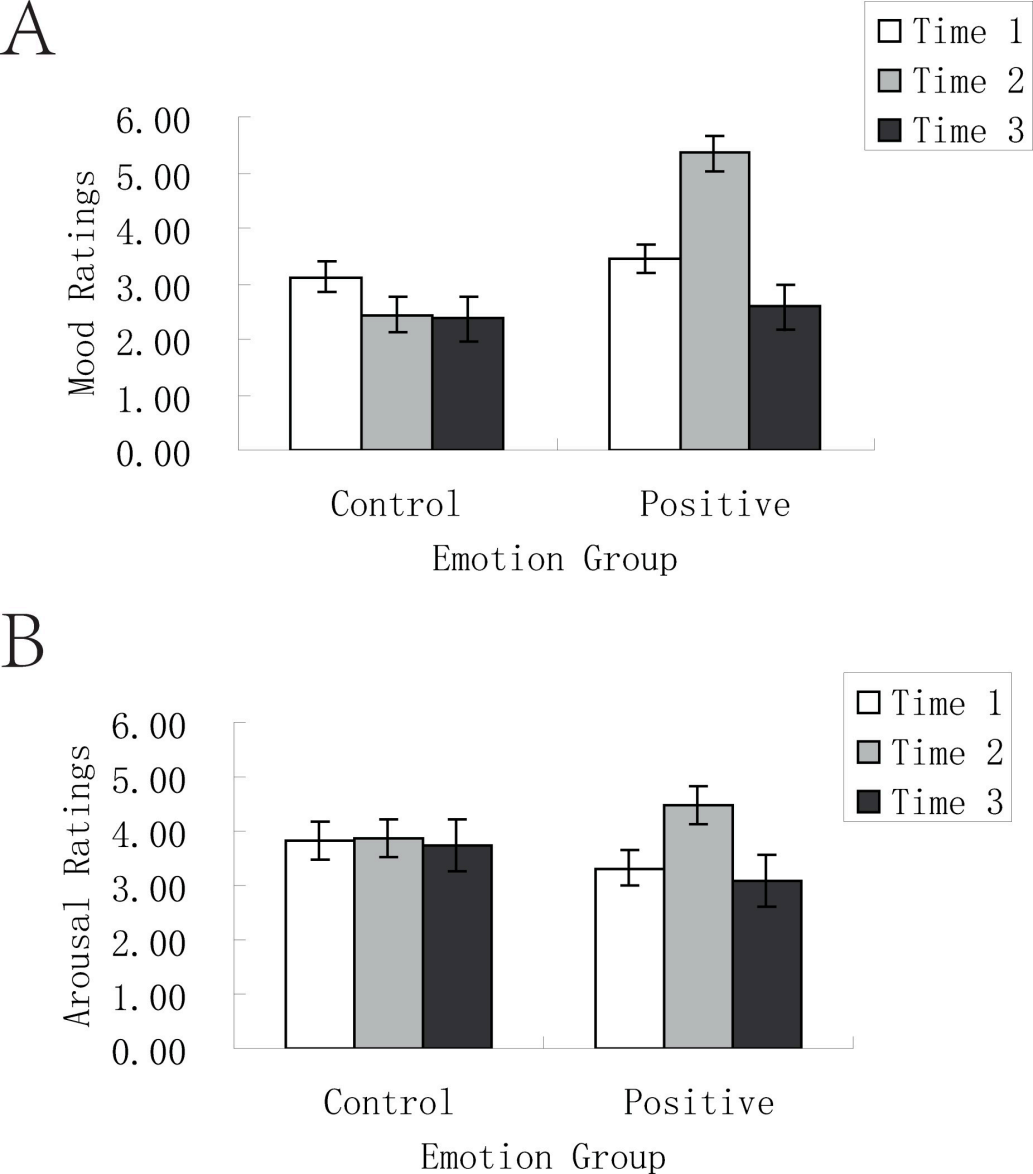
Mood and arousal ratings in the control and positive conditions in Experiment 1. (A) For participants in the control condition, mood ratings before video presentation, after video presentation and before delayed test did not significantly differ. For participants in the positive condition, mood ratings after video presentation was significantly higher than before video presentation; mood ratings before delayed test was significantly lower than before video presentation. (B) For participants in the control condition, arousal ratings before video presentation, after video presentation and before delayed test did not significantly differ. For participants in the positive condition, arousal ratings after video presentation was significantly higher than before video presentation; arousal ratings before delayed test did not significantly differ from that before video presentation.

*Manipulation check on emotion elicitation*. The ANOVA on mood ratings showed a significant main effect of time, *F* (1, 54) = 6.32, *p* = .015, η_p_^2^ = .11, and a significant main effect of emotion group, *F* (1, 54) = 23.45, *p* < .001, η_p_^2^ = .30. However, these main effects were qualified by a significant interaction between time and emotion group (Fig 1A), *F* (1, 54) = 27.45, *p* < .001, η_p_^2^ = .34. Participants in the control group had significant decrease in mood ratings over time, *t* = 2.17, *df* = 26, *p* = .039, cohen’s *d* = .85. Participants in the positive condition experienced significant increase in mood ratings over time, *t* = 5.05, *df* = 28, *p* < .001, cohen’s *d* = 1.91.

The ANOVA on arousal ratings showed a significant main effect of time, *F* (1, 54) = 8.18, *p* = .006, η_p_^2^ = .13, and a non-significant main effect of emotion group, *F* (1, 54) = .02, *p* = .88, η_p_^2^ < .001. Importantly, there was a significant interaction between time and emotion group (Fig 1B), *F* (1, 54) = 7.20, *p* = .01, η_p_^2^ = .12. Participants in the control group did not undergo significant change in arousal ratings over time, *t* = .12, *df* = 26, *p* = .91, cohen’s *d* = .06, whereas participants in the positive condition experienced significant increase in arousal ratings over time, *t* = 4.05, *df* = 28, *p* < .001, cohen’s *d* = 1.53.

*Initial performance before encoding*. The initial knowledge scores in the control group (*M* = .002, *SE* = .007) and positive group (*M* = .013, *SE* = .007) did not significantly differ, *F* (1, 54) = 1.11, *p* = .30, η_p_^2^ = .02. One sample *t* tests were conducted to check whether participants had already known the definitions of the English vocabulary before the learning phase. The results showed that, for participants both in the control and positive groups, the initial performance did not significantly differ from zero, *t* = 1.00, *df* = 26, *p* = .33, and *t* = 1.36, *df* = 28, *p* = .18, respectively. Furthermore, the ANOVA incorporating gender as a factor showed a non-significant main effect of gender, *F* (1, 52) = 1.22, *p* = .27, η_p_^2^ = .02. The interaction between gender and emotion group was not significant, either, *F* (1, 52) = 2.53, *p* = .12, η_p_^2^ = .05.

*Effect on associative memory*. The scores in the immediate test for the control and positive groups were .27 (*SE* = .04) and .33 (*SE* = .03), respectively. The scores in the delayed test for the control and positive groups were .25 (*SE* = .04) and .27 (*SE* = .04), respectively. An independent *t* test conducted on immediate memory scores indicated that the two emotion groups did not significantly differ, *t* = 1.37, *df* = 54, *p* = .18, cohen’s *d* = .37.

**Fig 2:**
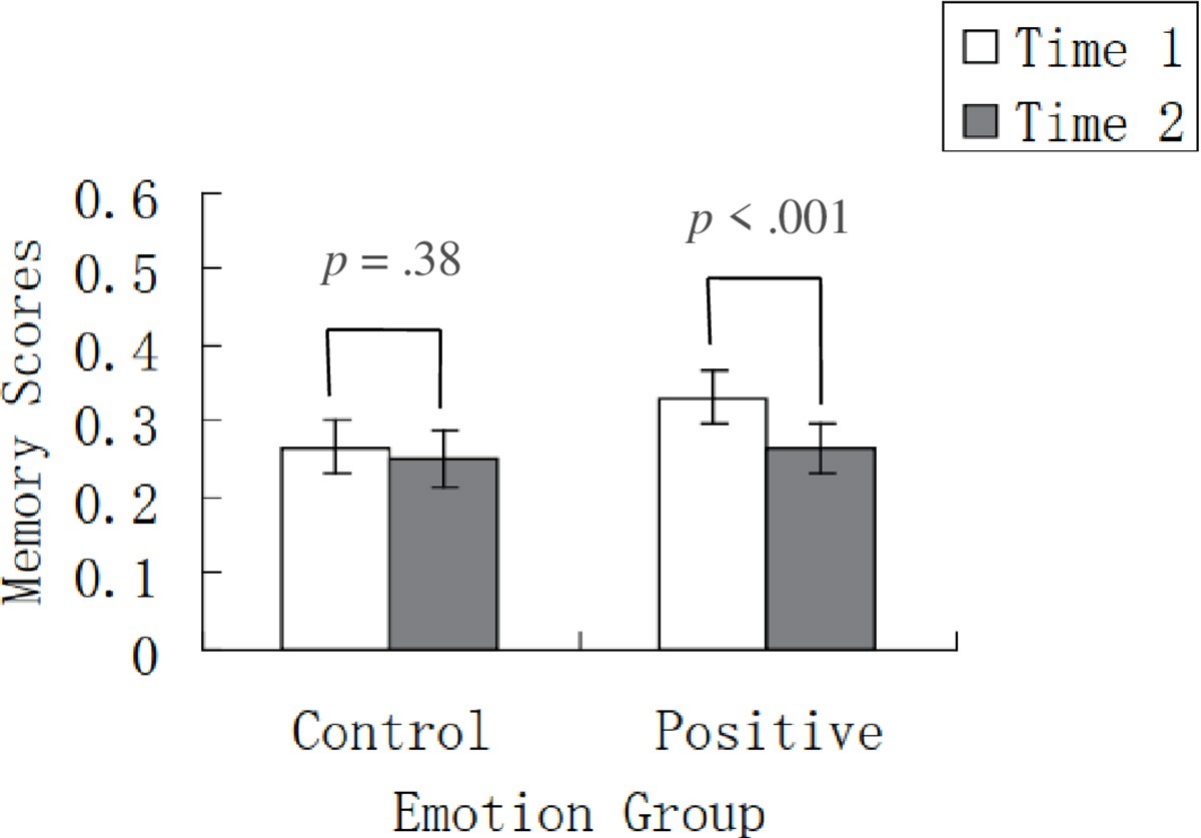
Memory performance in the control and positive conditions in Experiment 1. For the control group, memory scores in the delayed test did not significantly differ from those in the immediate test; for the positive group, memory scores in the delayed test was significantly lower than those in the immediate test. Time 1 and time 2 represent immediate test and delayed test, respectively. Error bars represent standard errors.

The ANOVA on scores in the immediate and delayed tests showed a significant main effect of time, *F* (1, 54) = 13.91, *p* < .001, η_p_^2^ = .21, indicating that memory scores were lower in the delayed test than in the immediate test. The main effect of emotion group was not significant, *F* (1, 54) = .67, *p* = .42, η_p_^2^ = .01. Importantly, there was a significant interaction between time and emotion group (Fig 2), *F* (1, 54) = 5.98, *p* = .018, η_p_^2^ = .10. Simple effect analyses further revealed that, for the control group, memory scores in the delayed test did not significantly differ from those in the immediate test (*p* = .38); for the positive group, memory scores in the delayed test was significantly lower than those in the immediate test (*p* < .001).

It is important to note that, prior to the final memory test, the control group (*M* = 2.37, *SE* = .42) and the positive group (*M* = 2.59, *SE* = .40) did not significantly differ in mood ratings, *t* = .37, *df* = 54, *p* = .71, cohen’s *d* = .11. In addition, the control group (*M* = 3.74, *SE* = .49) and the positive group (*M* = 3.10, *SE* = .47) did not significantly differ in arousal ratings, *t* = .93, *df* = 54, *p* = .36, cohen’s *d* = .26. Therefore, any differences observed in memory decrease could not be attributed to differences in mood and arousal before video presentation.

Considering that the immediate scores in the positive group were numerically higher than those in the control group, we conducted an ANOVA on the delayed memory scores, with the immediate memory performance being the covariate. The results showed that the effect of emotion group was significant, *F* (1, 53) = 4.97, *p* = .03, η_p_^2^ = .09, with worse delayed memory in the positive condition (*M* = .24, *SE* = .02) than in the control condition (*M* = .28, *SE* = .02).

One issue worth mentioning is that, after checking the data, we found that there were three (out of 56 in the final analysis) participants whose initial test scores exceeded zero: Two female participants in the positive condition whose initial test scores were 0.25 and 0.125, and one male participant in the control condition whose initial test score was 0.0625. This means that, in the positive condition, one female participant had already known the definitions of four English words (i.e., frisbee, hedgehog, emerald, rhinoceros), and another female participant had already known the definitions of only two English words (i.e., fennel, loquat); in the control condition, one male participant had already known the definition of only one English word (i.e., loquat). For all other participants, initial test scores were zero. We conducted further analyses using two approaches. With the first approach, we conducted a 2 (time: immediate vs. delayed) × 2 (emotion group: control vs. positive) repeated-measures ANOVA on the data excluding the data of the three participants. The result still showed a significant interaction between time and emotion group, *F* (1, 51) = 4.51, *p* = .039, η_p_^2^ = .08. Simple effect analyses revealed that, for the control group, immediate and delayed scores did not significantly differ (*p* = .38); for the positive group, however, delayed scores were significantly lower than immediate scores (*p* < .001).

With the second approach, we adjusted the scores of the above mentioned three participants by excluding their answers to the English words whose definitions they had already known in the initial test. Then we conducted a 2 (time: immediate vs. delayed) × 2 (emotion group: control vs. positive) repeated-measures ANOVA on all the data into which the above adjusted data were incorporated. The result still showed a significant interaction between time and emotion group, *F* (1, 54) = 5.83, *p* = .019, η_p_^2^ = .098. Simple effect analyses indicated that, for the control group, delayed scores did not significantly differ from immediate scores (*p* = .38); for the positive group, however, delayed scores were significantly lower than immediate scores (*p* < .001). Taken together, these findings suggest that the data of those three participants do not change the pattern of results.

The ANOVA incorporating gender as a factor showed no significant main effect of gender, *F* (1, 52) = 1.80, *p* = .19, η_p_^2^ = .03. Furthermore, the interaction between time and gender, *F* (1, 52) = .15, *p* = .70, η_p_^2^ = .003, between time, gender and emotion group, *F* (1, 52) < .001, *p* = .99, η_p_^2^ < .001, and between gender and emotion group, *F* (1, 52) = .03, *p* = .87, η_p_^2^ = .001, were all non-significant.

It is worth noting that the stimuli contained words of a range of emotional valence (e.g., “tarantula” vs. “emerald”). Because of the potential for arousal manipulation to interact with the emotional content of the memoranda, we conducted an analysis based on the data after removing three emotional words (i.e., tarantula, encephalitis, emerald). The results still showed a significant main effect of time, *F* (1, 54) = 5.55, *p* = .022, η_p_^2^ = .093, indicating that memory scores were lower in the delayed test than in the immediate test. The main effect of emotion group was not significant, *F* (1, 54) = .42, *p* = .52, η_p_^2^ = .008. Importantly, there was still a significant interaction between time and emotion group, *F* (1, 54) = 4.52, *p* = .038, η_p_^2^ = .08. Simple effect analyses further revealed that, for the control group, memory scores at the delayed test (*M* = .28, *SE* = .04) did not significantly differ from those in the immediate test (*M* = .28, *SE* = .04) (*p* = .87); for the positive group, memory scores at the delayed test (*M* = .29, *SE* = .04) was significantly lower than those in the immediate test (*M* = .34, *SE* = .04) (*p* = .002). An ANOVA was also conducted on the delayed memory scores, with the immediate memory performance being the covariate. The results showed that the effect of emotion group was marginally significant, *F* (1, 53) = 3.86, *p* = .055, η_p_^2^ = .07, with worse delayed memory in the positive condition (*M* = .26, *SE* = .02) than in the control condition (*M* = .31, *SE* = .02).

## Experiment 2

Experiment 1 showed that post-encoding positive emotion impaired associative memory for English words. In order to conceptually replicate the findings from Experiment 1, we conducted Experiment 2 in which different study material and videos for emotion induction were used. Furthermore, in Experiment 2 the participants were primary school students.

### Ethics statement

Verbal informed consent was obtained from all the primary school students who participated in the experiment; it was also obtained from the school principal and head teachers of all participating classes. Furthermore, written informed consent was also obtained from participants’ parents or guardians. Prior to participation, they were informed of the procedure, duration, and reward of the research. They were also informed that the participation was voluntary and they could withdraw at any time during the experiment. Data were collected and analyzed anonymously. The experiment was exempt from ethical review from the Institutional Review Board of School of Sociology and Psychology at Central University of Finance and Economics.

### Method

#### Participants

A sample size estimation was calculated using G*Power Version 3.1.9.2 software. Using moderate parameters (power = 0.8, effect size f = 0.25), the analysis yielded an estimate of a sample size of 34. A total of 56 students (28 men and 28 female participants; mean age = 10.63 years, *SD* = .91 year) from a rural primary school participated in the experiment. This sample size yields a power of 0.95. All participants were native Chinese speakers who had learned English for about 2–2.5 years. 40 of them were left-behind children, living with relatives other than their parents (their parents didn’t live with them because they worked in urban China). The present experiment was conducted in conformity with the ethical standards proposed by the [34]. Prior to students’ participation in the experiment, they received an informed consent form whereby the procedure, duration, and reward of the research were clarified. They were also informed that the participation was voluntary and they could withdraw at any time during the experiment. In addition, all the data obtained could be used only under anonymization condition and only for research purpose. Finally, we received written consent from the school principal and head teachers of all participating class, and then from 56 parents or guardians of all the children participants.

#### Stimuli

Two pictures and their corresponding English words were used for the practice phase. A total of 12 pictures for common items in daily life and their corresponding English words were used for the formal learning and testing phases.

Two 2-minute video clips were used. The neutral one was a segment of the neutral video used in Experiment 1. The positive one was a collection of comic scenarios in which some kids displayed comic behaviors. For instance, in one scenario, a kid was trying to ride an animal but ended up falling into a wooden bucket of water. In the pilot study, among the five students (one male participant and four female participants) who watched the positive video, four students (one male and three female participants) reported that they laughed during the video presentation.

#### Design and procedure

Identical to Experiment 1, a between-subjects design was used, with emotion group (control, positive) as the independent variable. Participants were randomly assigned to the two emotion groups. Table 3 presents the experimental steps and their corresponding duration.

**Table 3:** The steps and their duration in Experiment 2.

Step	Content	Duration in seconds	Duration in minutes
1	Initial knowledge test	36	0.60
2	Practice block (2 English-Chinese pairs)	12	0.20
3	Practice test	9	0.15
4	Formal learning block	144	2.40
5	Immediate test	48	0.80
7	Pre-video mood and arousal ratings	8	0.13
8	Video presentation	120	2.00
9	Post-video mood and arousal ratings	8	0.13
10	Filler tasks (math task)	371	6.18
11	Pre-test mood and arousal ratings	8	0.13
12	Delayed test	48	0.80

The duration of the steps for tests (i.e., initial knowledge test, practice test, immediate test, and delayed test) and math task were based on estimations because each trial did end until participants made a response.

After arriving at the laboratory, participants reported their basic demographic information as well as their degree of interest in memorizing English words (on a five-point likert scale ranging from “least interested” to “most interested”). This provided us with the opportunity to assess whether the two groups differed in their interest, which may be a confounding variable.

Then participants took an initial knowledge test in which a total of 12 pictures along with four English words were presented on a computer screen. Participants were asked to choose a correct English word that matches the picture from the four choices. No feedback was given as to whether their choices were correct or not. The positions at which correct choices occurred were balanced such that 1/4 of the correct choices appeared respectively at the four positions (see S2 Appendix). The purpose of the initial test was to ascertain whether participants had already known the English words for these pictures.

Then participants went through a brief practice block so as to familiarize themselves with the encoding task. Afterwards they were presented with a list of 12 pictures along with their English words and were asked to memorize the English word for each picture. No mention was given as to the subsequent memory tests. In each trial, a fixation cross was presented for one second, followed by a picture along with its English word lasting for five seconds. To avoid the potential floor effect, each picture and its associated English word was randomly presented twice.

Following the encoding stage, participants took an immediate test for the 12 items previously encoded. In each trial, a blank screen appeared for one second, followed by a picture along with four English words (see S2 Appendix). Participants were instructed to try their best to retrieve and make a correct choice. The immediate test allowed us to evaluate whether participants had indeed learned effectively and to ascertain whether participants assigned into the two emotion groups had comparable baseline memory performance.

After the immediate test, participants rated their current mood and arousal respectively on a 9-point scale before watching a 2-minute neutral or positive video depending on random assignment. After video presentation, they rated again their current mood and arousal. Then they executed some addition and subtraction math tasks, which lasted for about three minutes. The purpose of the math tasks was to prevent them from rehearsing the previously encoded pictures and words.

Prior to the delayed memory test, which took place 10 minutes after the end of encoding, participants took a rest that lasted for about one minute and then rated their mood and arousal. Unlike the previous experiment, the shorter retention interval was used in order to reduce the possibility of a floor effect, especially given that participants’ baseline performance was relatively low. Although it was likely that, with such a short retention interval, the emotion induction would exert its effect on retrieval, the ratings of mood and arousal prior to the delayed test made it possible to ascertain whether participants had returned to emotional “normalcy” and whether participants in the two emotion groups were emotionally comparable. The stimuli in Experiment 2 were the same as those in Experiment 1.

#### Statistical analyses

All analyses were carried out using SPSS 13.0. The significance value was set at p < .05. Memory performance was determined as percentage correct (i.e., correctly recognized English words divided by the total number of encoded English words). Degrees of freedom were adjusted where equality of variances was violated.

#### Results

*Participants’ characteristics*. Table 4 presents characteristics of the control and positive groups. It can be seen that participants in the two emotion groups did not significantly differ in age, years of English learning, and interest in memorizing English words. Furthermore, the ratio of number of male participants to number of female participants in the control group did not significantly differ from that in the positive group.

**Table 4:** Participants' characteristics in the control and positive groups in Experiment 2.

Measure	Control (n = 28)		Positive (n = 28)		*p*
	*M*	*SD*	*M*	*SD*	
Age	11.75	1.14	11.50	0.58	.31
Years of English learning	2.46	0.51	2.50	0.58	.81
Interest in memorizing English words	2.75	1.21	2.82	0.98	.81
M/F	14/14		14/14		> .99

M/F represents the ratio of number of male participants to the number of female participants.

*Interest in memorizing English words*. A *t*-test showed that the control and positive group did not significantly differ in their interest, *t* = .24, *df* = 54, *p* = .81, cohen’s *d* = .06. Therefore, any difference observed in memory cannot be attributed to difference in the interest in memorizing English words.

**Fig 3:**
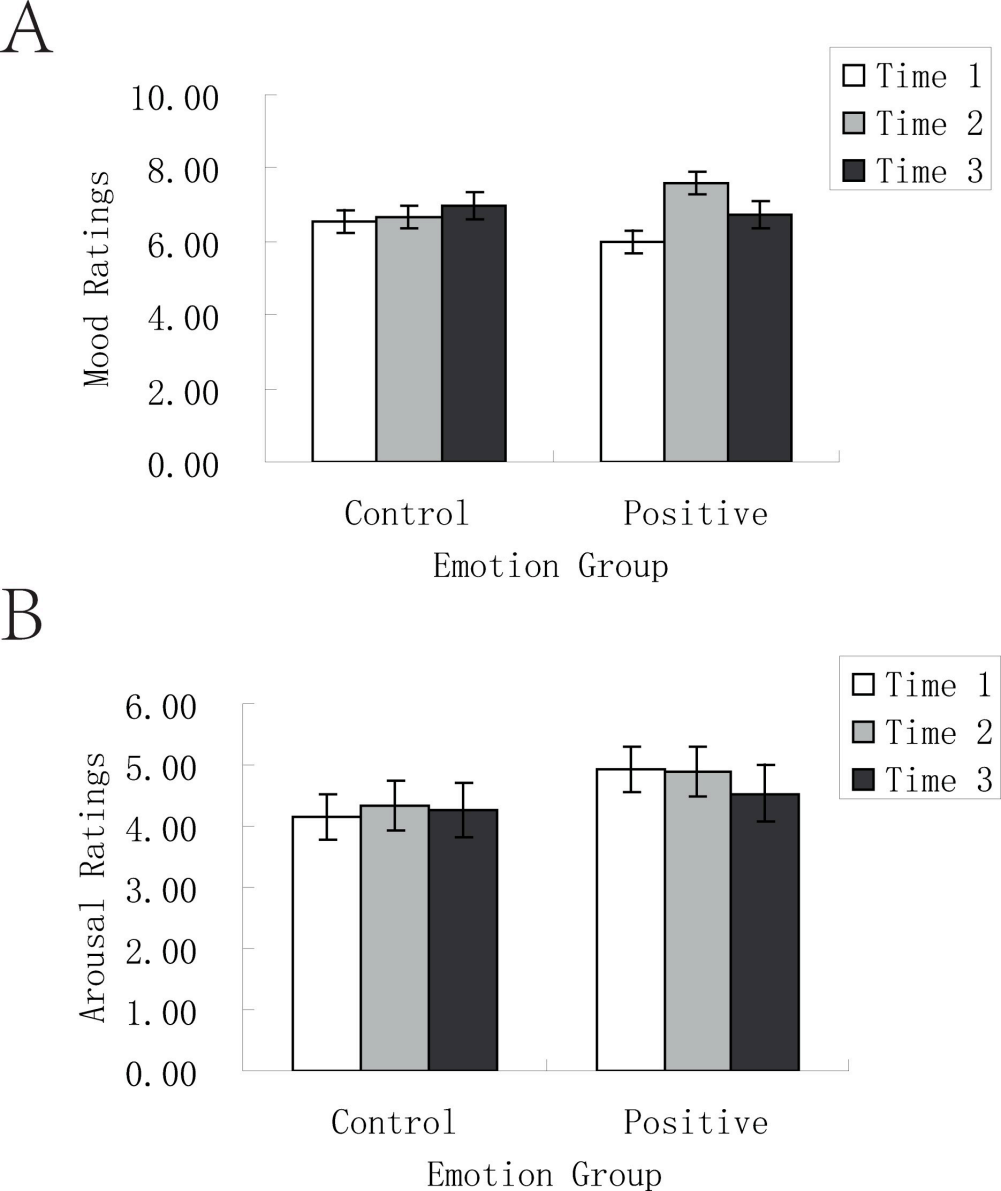
Mood and arousal ratings in the control and positive conditions in Experiment 2. (A) For participants in the control condition, mood ratings before video presentation, after video presentation and before delayed test did not significantly differ. For participants in the positive condition, mood ratings after video presentation and before delayed test were significantly higher than before video presentation. (B) Whether in the control condition or in the positive condition, arousal ratings before video presentation, after video presentation and before delayed test did not significantly differ. Time 1: before video presentation; time 2: after video presentation; time 3: before delayed test. Error bars represent standard errors.

*Manipulation check on emotion elicitation*. Table 3 presents mood and arousal ratings in the control and positive groups in Experiment 2. A 2 (time: before watching vs. after watching) × 2 (emotion group: control vs. positive) ANOVA on mood ratings showed a significant main effect of time, *F* (1, 54) = 11.63, *p* = .001, η^*2*^ = .18, which was quantified by a significant interaction between time and emotion group (Fig 3A), *F* (1, 54) = 8.96, *p* = .004, η^*2*^ = .14. Further analyses showed that participants in the control group did not have significant change in mood ratings over the time period, *t* = .28, *df* = 27, *p* = .79, cohen’s *d* = .11. Participants in the positive condition, however, experienced significant increase in mood over the time period, *t* = 4.92, *df* = 27, *p* < .001, with their mood ratings being significantly greater after video presentation than before video presentation. However, the ANOVA on arousal ratings showed neither significant main effects nor significant interaction between time and emotion group (Fig 3B) (all *p*s > .15).

*Initial performance before encoding*. The initial knowledge scores for the control and positive groups were .29 (*SE* = .02) and .30 (*SE* = .02), respectively. One sample two-tailed *t* tests showed a trend that the initial performance of the control group was above chance level (i.e., 0.25), *t* = 1.86, *df* = 27, *p* = .07. The initial performance of the positive group was significantly above chance level, *t* = 2.30, *df* = 27, *p* = .03. Nonetheless, there was no significant difference between the two groups regarding the initial knowledge scores, *t* = .09, *df* = 54, *p* = .93, cohen’s *d* = .03.

The ANOVA incorporating gender as a factor showed a non-significant main effect of gender, *F* (1, 52) = .08, *p* = .78, η_p_^2^ = .001. The interaction between gender and emotion group was not significant, either, *F* (1, 52) = .42, *p* = .52, η_p_^2^ = .008.

*Effect on associative memory*. The immediate scores for the control and positive groups were .40 (*SE* = .03) and .45 (*SE* = .03), respectively. The delayed scores for the control and positive groups were .42 (*SE* = .03) and .32 (*SE* = .03), respectively. The two emotion groups did not significantly differ in immediate memory scores, *t* = 1.35, *df* = 47.81, *p* = .18, cohen's *d* = .35.

**Fig 4:**
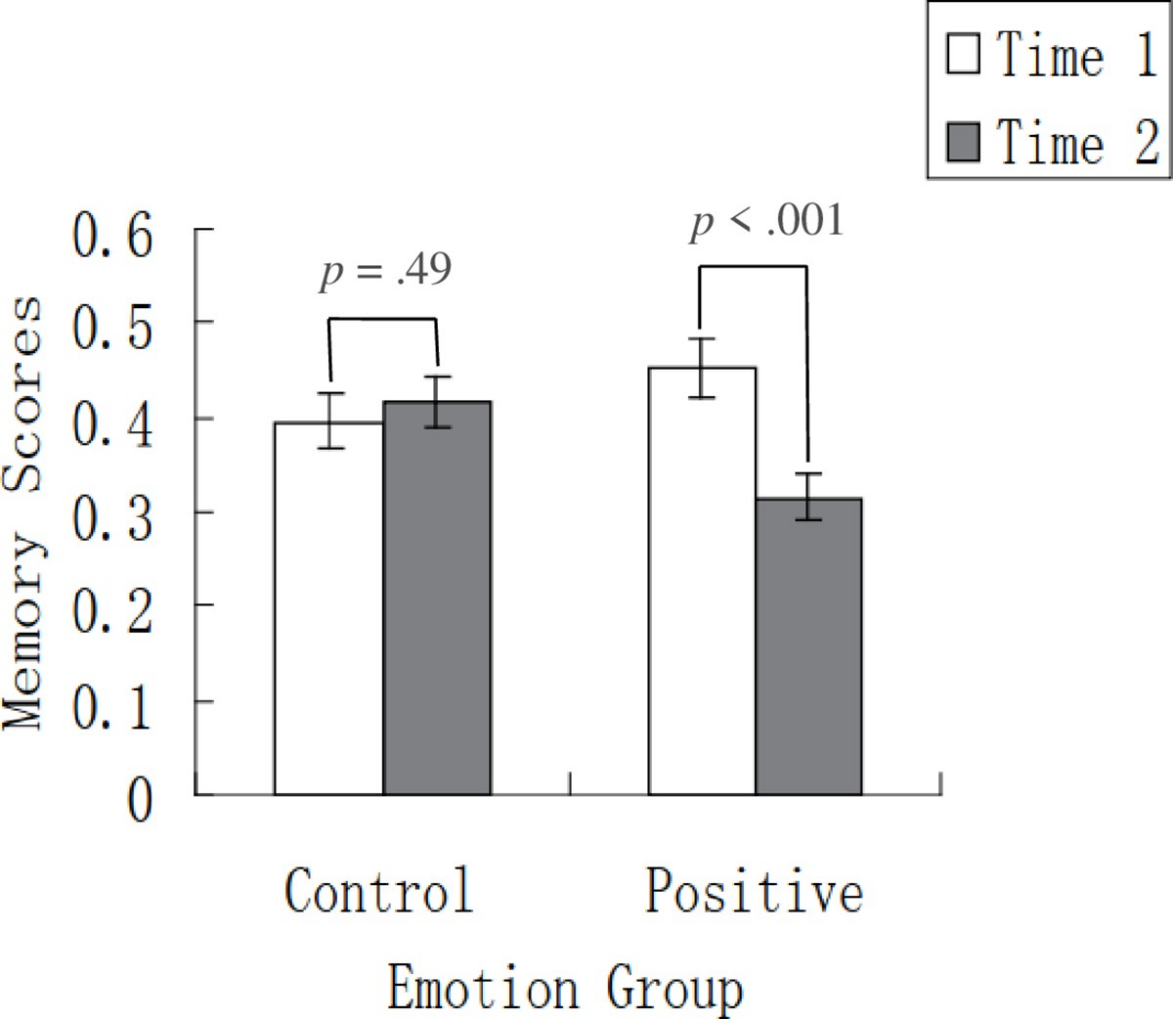
Memory performance in the control and positive conditions in Experiment 2. For the control group, memory scores in the delayed test did not significantly differ from those in the immediate test; for the positive group, memory scores in the delayed test was significantly lower than those in the immediate test. Time 1 and time 2 represent immediate test and delayed test, respectively. Error bars represent standard errors.

The ANOVA on scores in the immediate and delayed tests showed a significant main effect of time, *F* (1, 54) = 7.42, *p* = .009, η_p_^2^ = .12, indicating that memory scores were lower in the delayed test than in the immediate test. The main effect of emotion group was not significant, *F* (1, 54) = .46, *p* = .50, η_p_^2^ = .008. Importantly, there was a significant interaction between time and emotion group (Fig 4), *F* (1, 54) = 13.70, *p* = .001, η_p_^2^ = .20. Simple effect analyses further revealed that, for the control group, memory scores in the delayed test did not significantly differ from those in the immediate test (*p* = .49); for the positive group, memory scores in the delayed test was significantly lower than those in the immediate test (*p* < .001).

Considering that the immediate scores in the positive group were numerically higher than those in the control group, we conducted an ANOVA on the delayed memory scores, with the immediate memory performance being the covariate. The results showed that the effect of emotion group was significant, *F* (1, 53) = 12.66, *p* = .001, η_p_^2^ = .19, indicating worse delayed memory in the positive condition (*M* = .31, *SE* = .02) than in the control condition (*M* = .43, *SE* = .02). The above result corroborates those based on the repeated-measures ANOVA, suggesting the robustness of the impairing effect of post-encoding positive emotion.

The ANOVA incorporating gender as a factor showed that the main effect of gender was non-significant, *F* (1, 52) = .89, *p* = .35, η_p_^2^ = .02. In addition, the interaction between time and gender, *F* (1, 52) = 1.08, *p* = .30, η_p_^2^ = .02, between time, gender and emotion group, *F* (1, 52) = .04, *p* = .84, η_p_^2^ = .001, and between gender and emotion group, *F* (1, 52) = .16, *p* = .69, η_p_^2^ = .003, were all non-significant.

It is worth noting that, prior to the delayed test, the control group (*M* = 7.00, *SE* = .37) and the positive group (*M* = 6.71, *SE* = .37) did not significantly differ in mood ratings, *t* = .55, *df* = 48.82, *p* = .58. cohen's *d* = .16. Furthermore, the control group (*M* = 4.25, *SE* = .45) and the positive group (*M* = 4.54, *SE* = .45) did not significantly differ in arousal ratings, *t* = .45, *df* = 54, *p* = .66, cohen’s *d* = .13. Therefore, any differences observed in memory decrease could not be attributed to differences on emotional states before the delayed test.

## Discussion

The purpose of the current study was to investigate the effect of post-encoding positive emotion on associative memory. The results from Experiment 1 showed that post-encoding positive emotion impaired associative memory for high school students. Importantly, this impairing effect was replicated for children in Experiment 2, which differed from Experiment 1 in memory task, retention interval, video for induction of positive emotion, and the place for data collection. Taken together, the current findings support the hypothesis that, at least within a relatively short retention interval (i.e., up to 25 minutes), positive emotion induced after encoding can impair associative memory.

### Positive emotion impairs associative memory

Despite the general enhancement effect of positive emotion on learning of English as a foreign language (e.g., Dewaele & Alfawzan, 2018 [35–38]), the current study suggests that positive emotion is not universally beneficial. The impairing effect of positive emotion on memory for English vocabulary resonates with the studies showing the impairment effect of positive emotion [e.g., 39] and the studies showing that emotion, elicited during encoding by to-be-remembered stimuli, impairs associative memory [e.g., 13,14,16]. In accord with prior studies [e.g., 2,20,40], emotion in the current study was elicited by video clips that are independent of the memoranda, suggesting that the impairing effect of positive emotion can emerge regardless of temporal and semantic separation between source of elicitation and memorized stimuli.

There may be several explanations for the current findings. First, given prior studies showing an enhancing effect of post-encoding emotion on item memory [41], the impairing effect here might be due to the different mechanisms underlying item memory and associative memory, as proposed by the dual processing model of memory [e.g., 42]. For instance, [43] found greater prefrontal, hippocampal, and parietal activation for associations but not for items during encoding. Furthermore, they found differential neural mechanisms underlying memory retrieval: For item trials, greater activation was observed in bilateral frontal regions, bilateral anterior medial temporal areas, and the right temporo-parietal junction; for associative trials, however, the activation was found in the left dorsolateral prefrontal cortex and superior parietal lobules bilaterally. Consistent with [43], other researchers have found the important role of the hippocampus in associative memory [e.g., 44–46]. Therefore, although post-encoding emotion may enhance item memory through the amygdala-dependent processes, it may also impair associative memory via disruption to processing in the hippocampus [13].

The second explanation is that the retention interval in the current study was too short. This account receives support from the study by [47], who found that negative emotion impaired associtive memory under a short retention interval but enhanced it under a long retention interval (i.e., one week). It is likely that an enhancement effect would occur with an extended retention interval. Indeed, a recent study by [48] showed that post-encoding reward, which presumably induced positive emotion, retroactively enhanced memory for conceptually related information at a 24-h test but not at an immediate test. Importantly, the pattern of results from the above study was replicated by [49], who found that the effect of post-encoding reward emerged only at a memory test conducted 24 hours after encoding.

Third, the current result (particularly in Experiment 1) may be explained from a “priority” perspective. According to [50], arousal leads to the activation of the locus coeruleus-norepinephrine (LC-NE) system, which, in turn, enhances the processing of information of higher priority, regardless of information valence. They also stressed the importance of high behavioral activation (e.g., novelty-seeking), which may recruit the dopaminergic (DA) system to enlarge the scope of information processing.

In the current study, although we instructed participants to endeavor to associate Chinese definitions with English words, it is likely that this task were of high priority to participants. Furthermore, during the encoding task, participants could not be in a state of behavioral activation, making it difficult for them to integrate multiple event aspects (i.e., English words and their corresponding Chinese definitions) into memory.

Findings from the current study do not align with the evidence from past research showing that the effect of positive emotion is not pronounced. For instance, [2] found that post-encoding negative emotion, rather than positive emotion, affected item memory. In another study by [17], after encoding a list of neutral, positive, and negative words, participants took memory tests at one of eight delays (i.e., 0-min, 19-min, 63-min, 4.9-h, 8.75-h, 1-day, 6-day, and 14-day). They found enhanced free recall for negative but not positive words; furthermore, whereas negative words impaired recognition, no effect of positive emotion was observed. These effects of negative emotion dovetail with the NEVER (i.e., negative emotional valence enhances recapitulation) model proposed by [51], according to which negative events relative to positive events lead to enhanced encoding of sensory detail and create greater similarity between the encoding and retrieval signatures. Although the NEVER model has received support from a number of studies [e.g., 52,53], it seems to be inadequate to explain the impairing effect of positive emotion on associative memory.

In Experiment 1, participants filled out affective questionnaires following encoding rather than engaging in quiet rest. These questions may have interacted with the valence of the videos to influence memory. It must be noted, however, that using affective questionnaires as filler tasks has not been uncommon in prior studies [e.g., 32,42,54]. Importantly, in the current study, participants were randomly assigned to the control and positive groups and all participants performed exactly the same affective questionnaires during the waiting period. Therefore, any effect on memory cannot be attributed to filling questionnaires. In the current study we did not use quiet rest because we wanted to reduce the possibility that participants would conduct memory rehearsal, which is likely to occur in quiet rest. In addition, there has been evidence showing that quiet rest can contribute to memory consolidation [e.g., 55]. Consequently, using quiet rest might render it difficult to know whether any effect on memory comes from post-encoding emotion or from post-encoding rest.

### Post-encoding versus pre-retrieval effect

In many prior studies that used the paradigm of post-encoding emotion induction, delayed memory test was conducted 24 hours or 1 week after the end of encoding [e.g., 20,56,57]. Consequently, those studies are able to reveal the effect of emotion on time-dependent memory consolidation [e.g., 58]. In the current study, because the retention interval was rather short particularly in Experiment 2, it is unclear whether the impairing effect on associative memory is due to actions on consolidation or retrieval processes. Even though the data showed that subjective emotion ratings did not significantly differ immediately before memory retrieval, due to the small temporal gap between emotion induction and delayed test, we are not in the position to disambiguate between a post-encoding or pre-retrieval effect.

However, both rodent and human studies have shown that the effect of emotion/stress is contingent upon the timing of induction (for a review, see Goldfarb et al., 2019 [59]). Specifically, although acute stress induced pre- and post-encoding has been found to enhance item and associative memory, there has been mounting evidence that acute stress impairs memory retrieval [e.g., 60,61]. Such adverse effects may be attributed to the influence of stress-induced glucocorticoids on brain regions such as the hippocampus [60] and the prefrontal cortex [62]. Furthermore, stress may exert its negative effects on memory via suppressing processing of information unrelated to the stressor. In the current study, the impairing effect of positive arousal might have arisen from the disruption to memory retrieval as a result of the disruption to the brain regions such as the hippocampus and prefrontal cortex, the latter of which has been assumed to be involved in free recall and cued-recall tasks [61].

### Contributions

The current study extends prior research in several aspects. First, although post-encoding emotion has been shown to enhance item memory for pictures [25,63], words [57,64,32,40,20,56,2] and college examination performance [65], the current study is the first to provide the evidence that post-encoding positive emotion can decrease associative memory, thus suggesting the effect of emotion on item memory does not necessarily extend to that on associative memory.

Second, the current study extends prior studies by providing the first evidence that the impairing effect of emotion can extend to kids around 10 years old. This suggests that, when it comes to the effect of post-encoding positive emotion, the mechanism to which children are subject may overlap that to which adults are subject. Although no neuroimaging data were collected, children may have similar activation of the amygdala in response to positive emotion, given the evidence showing that even children (8–16 years of age) can have amygdala activation to emotional stimuli [e.g., 66]. That said, prior studies have indicated memory of children and adults can be differentially affected by emotional stimuli. For instance, [22] found higher memory accuracy for the negative than positive words in adolescents (12-14-year-olds). However, for children (7-8-year-olds), there was no influence of word valence on memory accuracy. In their study, however, emotion was not induced independently of the memoranda. These findings suggest that whether children and adults can be similarly affected may be contingent on the memory stage during which emotion is manipulated.

### Limitations

The current study has at least three limitations. First, the control and positive groups differed in performance in the immediate memory test, although the difference did not reach significance. Indeed, it is possible that participants in the positive group had greater decrease in memory because their baseline performance was higher and as such had more to forget. However, the analyses in which the baseline performance was included as a covariate suggest that reduction in associative memory for participants in the positive condition might not be attributed to their numerically higher baseline performance.

Second, there are some problems with the videos used to induce emotion. Specifically, in Experiment 2, participants who watched the comic video did not undergo significant increase in arousal ratings, although they did have significant increase in mood ratings. This finding may detract from the conclusion that positive emotion can impair associative memory. However, 22 out of the 28 participants assigned to the positive group reported bursting into laughters. Furthermore, the mean times of laughter for the 22 participants reached 4.9 times (*SE* = .53). This indicates that most of them were actually highly aroused even though they did not provide corresponding arousal ratings. Nonetheless, an ideal approach in future studies would be to collect both subjective reports and physiological data such as heart rate and skin conductance so as to better evaluate the induction effectiveness in terms of arousal. Furthermore, although the video clips were successful in the sense that the positive video, relative to the neutral video, significantly enhanced both mood and arousal ratings in Experiment 1 and significantly enhanced mood ratings in Experiment 2, other factors might have affected memory because the two videos differ in a number of aspects, such as presence of humans, informational content and attentional engagement. Indeed, whereas no person was present in the neutral video, three persons (i.e., comedians) were shown in the positive video. In addition, the script for the positive video consists of 748 words, whereas the script for the neutral video consists of 421 words. Additionally, participants who watched the positive video could have greater attentional engagement during video presentation although we did instruct participants to watch carefully without looking away. Therefore, it is possible that the memory impairment has occurred due to the positive video creating more retroactive interferences. Caution, must be exercised in interpreting the current findings from a valence perspective. That said, in prior studies that employed the same neutral and positive videos [e.g., 54], positive emotion was observed to enhance recognition memory performance, which suggests that retroactive interferences per se may not be responsible. Furthermore, because the content of to-be-remembered materials (i.e., definitions of English vocabulary) does not overlap that of both the neutral and positive videos, retroactive interferences are less likely.

It should be noted that creating a positive video that matches a neutral video in a variety of dimensions can be a difficult task and, indeed, few researchers have effectively addressed this issue. For instance, in most prior studies [e.g., 54], participants in the neutral and positive groups were respectively presented with a video on CD-ROM repairing and a comic video, which differ in a variety of aspects. That said, in order to draw a solid conclusion on the role of emotional valence, varying video clips should be used in future research. Furthermore, efforts need to be made in future studies to minimize the possibility that videos used in the neutral and positive conditions differ in dimensions other than valence.

Third, there has been evidence that stimuli of negative valence, rather than positive valence, produce deficits in associative memory [e.g., 47]. In the current study we only induced positive emotion, rendering it difficult to determine whether the observed effects are valence specific. With that being said, it is important to note that to date few studies have examined the effect of positive versus negative emotion induced after encoding. Indeed, in the majority of prior studies only negative emotion was elicited via a video clip on dental surgery [e.g., 56]. In the studies that did induce both positive and negative emotions, the memory task did not tap associative memory (e.g., 25,57). Future studies, therefore, are needed to elucidate whether the effect of post-encoding emotion on associative memory is contingent on emotional valence.

### Theoretical and practical implications

Findings from the current study have both theoretical and practical implications. For previous studies showing the beneficial role of post-encoding positive emotion on memory [20,64], the theoretical account posits that emotion leads to release of a variety of substances including norepinephrine, which, along with the activation of the amygdala, modulates hippocampus-dependent memory [19]. The current findings suggest that this account does not explain the impairment effect of post-encoding emotion on associative memory. The current study provides the caveat that positive emotion induced after encoding is not ubiquitously beneficial for memory and highlights the need to research for the boundary conditions under which emotion respectively impairs and enhances item vs. associative memory.

The findings on the impairing effect of post-encoding positive emotion (activated after English vocabulary learning) on associative memory for English vocabulary also provide pedagogical implications for English (L2) vocabulary teaching and learning. Specifically, under the context of globalization, having a good mastery of English has become increasingly critical, which is particularly the case for the majority of students in China, where English is compulsory from primary to tertiary education level [67]. It is apparent that a good memory for vocabulary is a prerequisite for mastery of English because “the heart of language comprehension and use is the lexicon” [68]. The current study shows that watching a laughter-provoking video clip after English vocabulary learning reduced associative memory for English words, suggesting that English teachers should be cautious in drawing on positive emotion intervention as teaching strategy [e.g., humor in L2 teaching in the study of 69–71] in classroom practices. Specifically, after the learning of English vocabulary, it may behove a learner to be emotionally neutral. However, according to the large body of studies in second/foreign language acquisition/learning, positive emotions are central to second language learners’ learning performance and achievement [e.g., 35–37, 72–76]. Thus, future studies need to further investigate the conditions under which positive emotion exerts impairing and enhancing effects, respectively.

## Supporting information

S1 Appendix The English words and the corresponding Chinese definitions used in Experiment 1. (DOC)

S2 Appendix The picture names and the four choices from which participants were instructed to select a correct one. The bold words represent the correct ones.
(DOCX)

S1 Data (RAR)
