# Supplementary material for: Post-encoding positive emotion impairs associative memory for English vocabulary
Source: PLoS One. 2020 Apr 6;15(4):e0228614. doi: 10.1371/journal.pone.0228614 (PMC7135307; doi:10.1371/journal.pone.0228614)
Supplement: S1 Appendix — (DOC) [file pone.0228614.s001.doc]

**Appendix I .**

The English words and the corresponding Chinese definitions used in Experiment 1.

| NO. | English Words | Chinese definitions |
| --- | --- | --- |
| 1 | pancreas | 胰腺 |
| 2 | emerald | 绿宝石 |
| 3 | hedgehog | 刺猬 |
| 4 | stethoscope | 听诊器 |
| 5 | albumen | 蛋清 |
| 6 | frisbee | 飞盘 |
| 7 | encephalitis | 脑炎 |
| 8 | fennel | 茴香 |
| 9 | tarantula | 狼蛛 |
| 10 | emetic | 催吐剂 |
| 11 | rhinoceros | 犀牛 |
| 12 | xylophone | 木琴 |
| 13 | platypus | 鸭嘴兽 |
| 14 | peninsula | 半岛 |
| 15 | biotite | 黑云母 |
| 16 | loquat | 枇杷 |

Note. The selection of words was primarily based on two criteria: 1) novelty (i.e., the words are new to participants so as to allow for the examination of the effect of emotion); 2) single definition (i.e., the words have single definitions rather than multiple definitions).
