# Supplementary material for: Post-encoding positive emotion impairs associative memory for English vocabulary
Source: PLoS One. 2020 Apr 6;15(4):e0228614. doi: 10.1371/journal.pone.0228614 (PMC7135307; doi:10.1371/journal.pone.0228614)
Supplement: S2 Appendix — The bold words represent the correct ones. (DOCX) [file pone.0228614.s002.docx]

**Appendix II**

The picture names and the four choices from which participants were instructed to select a correct one. The bold words represent the correct ones.

| Picture Name | Choice 1 | Choice 2 | Choice 3 | Choice 4 |
| --- | --- | --- | --- | --- |
| antenna | **1. antenna** | 2. antenatal | 3. antelope | 4. anthem |
| axe | **1. axe** | 2. axis | 3. axel | 4. axion |
| basket | **1. basket** | 2. bask | 3. basis | 4. basin |
| bathtub | 1. bathe | **2. bathtub** | 3. bathwater | 4. bathhouse |
| chimney | 1. chimra | **2. chimney** | 3. chimenea | 4. chime |
| couch | 1. cough | **2. couch** | 3. could | 4. council |
| peacock | 1. peachy | 2. peace | **3. peacock** | 4. pecker |
| kettle | 1. kernal | 2. ketch | **3. kettle** | 4. kestrel |
| rocket | 1. rocketry | 2. rocker | **3. rocket** | 4. rocky |
| pumpkin | 2. pump | 2. pumice | 3. pummel | **4. pumpkin** |
| fence | 1. fend | 2. fencer | 3. fennel | **4. fence** |
| hanger | 1. hangar | 2. hang | 3. hangover | **4. hanger** |
